# Dynamics of the gut microbiome and metabolome in children during and after an infectious diarrheal episode

**DOI:** 10.3389/fcimb.2026.1905670

**Published:** 2026-08-27

**Authors:** Daniel Melipil-Millán, Mariana Izquierdo, Roberto Ovalle, Francisca A. Espinoza-Cavieres, Pablo Gallardo, Romina Valenzuela, Mauricio J. Farfan

**Affiliations:** 1 Departamento de Cirugía y Pediatría Oriente, Centro de Investigación Clínica Avanzada (CICA) Hospital Dr. Luis Calvo Mackenna, Facultad de Medicina, Universidad de Chile, Santiago, Chile; 2 Groningen Institute for Evolutionary Life Sciences (GELIFE) – University of Groningen, Groningen, Netherlands

**Keywords:** acute diarrhea, children, diarrhea recovery, gut microbiota, short-chain fatty acids

## Abstract

**Introduction:**

Acute diarrhea is associated with significant alterations in the gut microbiota and metabolome. Despite several studies documenting increased levels of histamine and short-chain fatty acids (SCFAs) during diarrheal episodes, the dynamics of these metabolites and microbiota following clinical recovery remain poorly understood, especially in children.

**Methods:**

We evaluated the composition of the intestinal microbiota and the levels of histamine and SCFAs in stool samples from children under five years old with acute diarrhea and after clinical recovery. Additionally, a comparison with a sample of healthy children was included.

**Results:**

Our results revealed significant differences in the community structure of the intestinal microbiota among the three conditions. The recovery samples exhibited a greater abundance of genera associated with diarrhea, such as *Escherichia-Shigella, Streptococcus, Veillonella*, and *Akkermansia*, as well as genera associated with healthy microbiota, such as *Bifidobacterium*. Regarding metabolites, butyrate levels decreased following clinical recovery, reaching values comparable to those found in the healthy samples, whereas histamine levels did not differ significantly among the conditions.

**Discussion:**

Our findings suggest that, after clinical resolution of diarrhea, the gut microbiota and its metabolites remain significantly different from those observed in healthy children, possibly representing an intermediate stage between diarrhea and a healthy condition. These findings suggest that recovery of the gut microbiota to a healthy condition may take longer and does not necessarily coincide with clinical recovery from diarrhea.

## Introduction

Acute diarrhea remains a leading cause of morbidity and mortality among children under five years of age, particularly in low-income countries, and continues to impose a substantial global burden (Florez et al., 2020; Cohen et al., 2022; World Health Organization, 2024). Although etiological agents are diverse, worldwide viruses are estimated to account for ~70-90% of diarrheal cases, followed by bacteria (~10–20%), while parasites represent <5% (Florez et al., 2020; Poulain et al., 2021).

Multiple studies have demonstrated marked dysbiosis following diarrheal episodes, with reductions in richness and diversity compared to healthy children (Curtis et al., 2014; Singh et al., 2015; Dinleyici et al., 2018). Specifically, increases in Firmicutes and decreases in Proteobacteria have been reported in rotavirus-associated diarrhea (Dinleyici et al., 2018), and infections caused by *Campylobacter*, *Salmonella*, or *Shigella* (Singh et al., 2015). A similar trend has been observed in our samples, with increased Bacteroidetes in diarrhea caused by diarrheagenic *E. coli* (DEC) compared with healthy children (Gallardo et al., 2020). These microbiota alterations are intrinsically linked to the intestinal metabolome and host health. In a previous study, we identified modifications in the intestinal metabolome of children following DEC-associated diarrhea, including elevated levels of histamine and SCFAs (Gallardo et al., 2020; Gallardo et al., 2024). Collectively, these observations are consistent with impaired metabolite absorption and increased intestinal permeability during diarrheal episodes (de la Cuesta-Zuluaga et al., 2018), which may promote inflammation, hinder the restoration of homeostasis (Dinleyici et al., 2018), and even enhance the virulence of certain pathogens such as DEC (Izquierdo et al., 2022).

While the acute phase of diarrhea induces profound shifts in the gut microbiota and metabolome, the mechanisms underlying their restoration after clinical recovery are less well understood. Few studies have evaluated changes in microbiota after recovery from a diarrheal episode. In adults, reports indicate that microbial diversity after clinical recovery remains lower than during the acute diarrheal episode (Becker-Dreps et al., 2015; Hansen et al., 2024). However, another study found that, when comparing recovered patients with healthy family members, microbial compositions were similar between the samples regardless of pathogen type or patient age (Singh et al., 2015). Studies of diarrheal episodes in children have reported a recovery pattern in which alpha diversity is reduced after a diarrheal event, followed by a time-dependent increase, ultimately reaching higher levels than those observed during acute infection (Singh et al., 2015; Mortensen et al., 2018). From a metabolomic perspective, one study using metabolomic predictions demonstrated that diarrhea in adults is associated with shifts in the metabolic profile and lower metabolite diversity, whereas clinical recovery is associated with greater metabolome diversity (Hansen et al., 2024).

Although these studies evaluated metabolic and microbiota changes following recovery from diarrhea, paired studies in children that integrate microbiota dynamics with changes in diarrhea-associated metabolites during the diarrheal episode and after clinical recovery are lacking. In this work, we ask whether changes in the intestinal microbiota and metabolites associated with an episode of diarrhea are restored after recovery. To address this question, we conducted a prospective paired analysis examining gut microbial dynamics alongside fecal metabolites (histamine and SCFA) in children (<5 years old) during acute diarrheal illness and following clinical recovery.

## Materials and methods

### Study design and participants

A prospective descriptive study on diarrhea and recovery was conducted at the Dr. Luis Calvo Mackenna Hospital from June 2018 to October 2023. Children who presented to Dr. Luis Calvo Mackenna Hospital with acute diarrhea and tested positive for common diarrheal pathogens using a FilmArray^®^ GI panel were enrolled. The clinical course and treatment of the diarrhea, including antibiotic and probiotic use, were determined from the patients’ medical records. Written informed consent was obtained from legal guardians prior to enrollment. The Ethics Committee of the Faculty of Medicine at the University of Chile approved the study protocol (Approval No. 031-2017). After enrollment, a nurse contacted the parents or legal guardians to inquire about the children’s health condition. Once the children had recovered from the diarrhea, a new stool sample was requested. Recovery was defined as a general condition of health, without gastrointestinal discomfort, fever, vomiting, or diarrhea. Children whose medical records could not be obtained were excluded from the study.

Healthy children were selected from previously enrolled children from August 2017 to November 2020, and included those free of acute and chronic infections, not exposed to antibiotics, and with no history of diarrhea at least 1 month prior to sample collection (Gallardo et al., 2024). All the stool samples were negative for common diarrheal pathogens using the FilmArray^®^ GI panel. Written informed consent was obtained from legal guardians prior to enrollment. The Ethics Committee of the Faculty of Medicine at the University of Chile approved the study protocol (Approval No. 040-2016).

### Sample size calculation

Sample size was determined using G-Power (Faul et al., 2007). Considering a type I error of 0.05, a potency of 80%, and an effect size of 0.455, we calculated that a minimum of 51 samples were required, with 17 per condition (diarrhea, recovery, and healthy). The effect size was based on gut microbiota composition in a pilot study conducted in our laboratory, which compared 10 samples per condition.

#### Gut microbiota analysis

DNA was extracted from fecal samples using the DNeasy PowerSoil Pro Kit (Qiagen). The V3–V4 region of the 16S rRNA gene was amplified. Libraries were sequenced as follows: diarrhea/recovery samples were newly sequenced on an Illumina NextSeq 1000 (paired-end), whereas the healthy samples were reanalyzed using previously generated 16S datasets from a prior cohort sequenced on MiSeq v3 (2×300 bp) (Gallardo et al., 2020). Bioinformatic processing was performed in R using the DADA2 (v1.18.0) pipeline (Callahan et al., 2016), including quality filtering, paired-read merging, chimera removal, and inference of amplicon sequence variants (ASVs). Taxonomy was assigned using the SILVA v138 database (Mandal et al., 2015). Microbiota analysis was assessed with the phyloseq (McMurdie and Holmes, 2013) and vegan (v2.7-0) (Oksanen et al., 2025) packages in the R software.

#### Metabolite quantification

Fecal histamine levels were measured by enzyme-linked immunosorbent assay (ELISA) (*Histamine ELISA*, Immunodiagnostik AG), following the manufacturer’s instructions. For SCFAs (acetate, propionate, butyrate), high-performance liquid chromatography (HPLC) with UV detection was used, using a protocol adapted in the laboratory (Gallardo et al., 2020). Concentrations were expressed in μmol/g of the fecal sample. Samples below the detection limit were excluded.

#### Statistical analysis

Metabarcoding analysis was processed using R software (version 4.1.2). Alpha-diversity indexes (number of observed ASVs, Chao 1, Shannon, and InvSimpson index) were compared between the diarrhea and recovery samples using the Wilcoxon test. β-diversity was analyzed using unconstrained Principal Coordinate Analysis (PCoA), which showed the distribution of samples based on species-abundance similarities. Also, a constrained Redundancy Analysis (RDA) was performed to examine the distribution of samples, with the analysis conditions as the explanatory variable and species abundances as the response variable. Bray-Curtis distances were used for both analyses, and the enclosing ellipses were drawn based on a 95% confidence interval or the Khachiyan algorithm. Significance between samples was assessed with a PERMANOVA test, using a paired/design-aware analysis for the diarrhea and recovery comparisons.

Relative abundance analyses at the phylum and genus levels were made for those taxa that showed levels higher than 1% in the recovery samples. Statistical analysis for paired samples was performed using the Wilcoxon signed-rank test, and comparisons with the healthy samples were carried out using an ANOVA followed by Tukey’s *post-hoc* test. Likewise, comparisons of metabolite levels between the diarrhea and recovery samples were performed using the Wilcoxon signed-rank test, and comparisons between the diarrhea and healthy samples were performed using the Kruskal-Wallis test followed by Dunn’s *post-hoc* test. These analyses were conducted using GraphPad Prism 6. For all comparisons, statistical significance was set at p<0.05. To control Type I error due to multiple comparisons, p-values were adjusted using the Benjamini-Hochberg False Discovery Rate (FDR) procedure, with statistical significance set at q<0.05.

## Results

### Patient’s characteristics and pathogen detection in fecal samples

Twenty-three children were enrolled; the median (interquartile range; IQR) age was 26 (13–51) months, with no major differences in sex distribution or delivery type. Regarding clinical characteristics, most participants in both the diarrhea and healthy conditions were breastfed (87.0%). Most children reported no allergies and did not receive probiotic or antimicrobial treatment at enrollment. **Table 1** summarizes the baseline characteristics of the study participants in both the diarrhea and healthy groups.

**Table 1:** Characteristics and background of enrolled children.

Characteristics	Diarrhea group	Healthy group
	(*n* = 23*)*	(*n* = 23)
Sex	*n* (%)	*n* (%)
Male	15 (65.2)	11 (47.8)
Female	8 (34.8)	12 (52.2)
Type of delivery		
Vaginal	10 (43.5)	11 (47.8)
Cesarean	12 (52.2)	12 (52.2)
N/A	1 (4.3)	0 (0.0)
Breastfeeding		
No	1 (4.3)	3 (13.0)
Yes	20 (87.0)	20 (87.0)
N/A	2 (8.7)	0 (0.0)
Any types of allergies		
No	20 (87.0)	19 (82.6)
Yes	3 (13.0)	4 (17.4)
Probiotic treatment		
No	19 (82.6)	18 (78.3)
Yes	4 (17.4)	5 (21.7)
Antibiotic treatment		
No	18 (78.3)	23 (100.0)
Yes	5 (21.7)	0 (0.0)

In the diarrhea group, the median (IQR) of recovery sampling time was 2.5 (1.8-3.2) months. In diarrhea samples, a single bacterial detection was observed in 15/23 (65.2%) cases, a single viral detection in 3/23 (13.0%) cases, bacterial co-detection in 4/23 (17.4%) cases, and bacterial-viral co-detection in 1/23 (4.3%) cases. Recovery samples exhibited pathogen detection in 12/23 (52.2%) samples; a bacterial single detection was found in 2/23 (8.7%) samples, bacterial co-detections in 4/23 (17.4%) samples, viral single detection in 1/23 (4.3%) samples, and bacterial-viral co-detections in 5/23 (21.7%) samples (**Table 2**). Also, detailed information for each enrolled child with diarrhea, including the time between the diarrhea episode and recovery sample collection, use of antibiotics and probiotics for treatment of diarrhea, and other variables, is provided in **Supplementary Table 1**.

**Table 2:** Characteristics of diarrheal and recovery fecal samples in the diarrhea/recovery study.

Characteristics	Diarrhea samples	Recovery samples
	(*n* = 23)	(*n* = 23)
Age in months at sampling	*n* (IQR or %)	*n* (IQR or %)
Median (IQR)	26 (13-51)	28.5 (14.8-54.2)
Pathogen detection in samples		
1 bacterium	15 (65.2)	2 (8.7)
2 bacteria	4 (17.4)	4 (17.4)
1 virus	3 (13.0)	1 (4.3)
1 bacterium and 1 virus	1 (4.3)	3 (13.0)
2 bacteria and 1 virus	0 (0)	2 (8.7)
Negative for any pathogen	0 (0)	11 (47.8)

### SCFA and histamine levels

**Figure 1:**
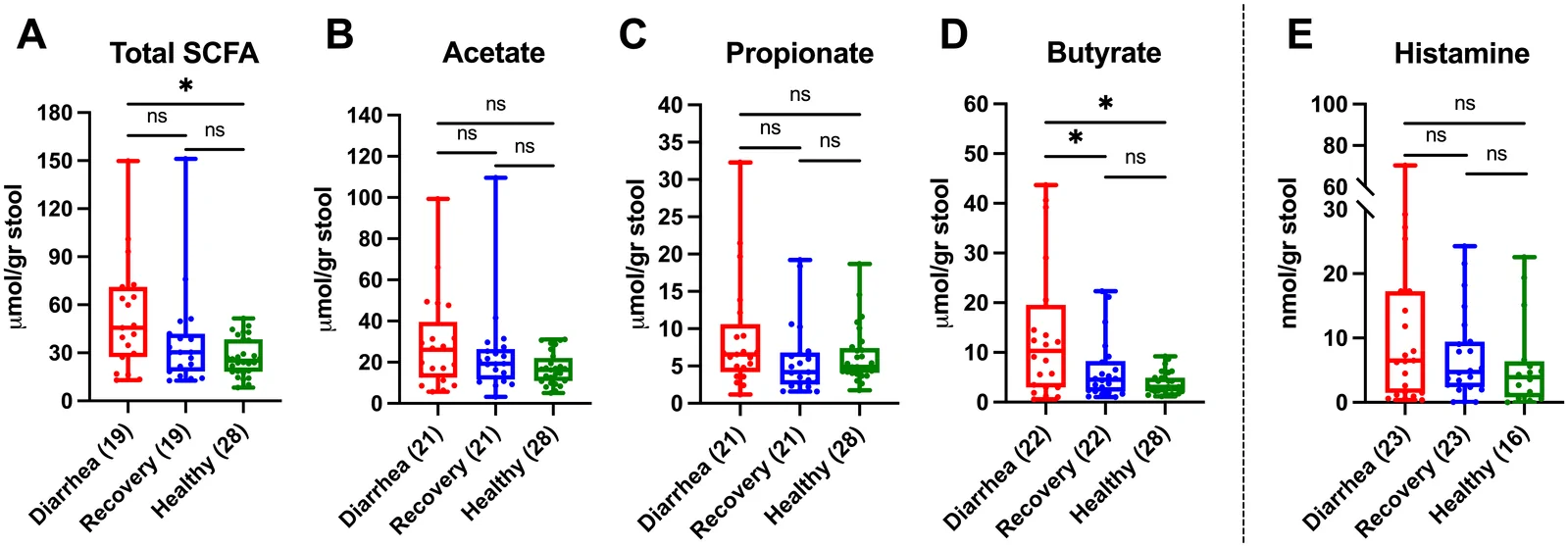
Total SCFA, acetate, propionate, butyrate, and histamine concentrations in the diarrhea, recovery, and healthy samples. Levels of total **(A)** SCFAs, **(B)** acetate, **(C)** propionate, **(D)** butyrate, and **(E)** histamine for the diarrhea (red), recovery (blue), and healthy (green) samples. Significant differences between the diarrhea and recovery samples were determined using the Wilcoxon matched-pairs signed-rank test, and comparisons with the healthy samples were made using the Kruskal-Wallis test followed by Dunn’s *post-hoc* test, with p-values adjusted for multiple comparisons using the False Discovery Rate (FDR). Significance is indicated (*p<0.05).

Total SCFA levels were higher in diarrheal samples than in healthy samples, but no difference was observed between diarrheal and recovery samples (**Figure 1A**). Acetate and propionate concentrations did not differ between the diarrhea and recovery samples, nor did they differ when each was compared with the healthy samples (**Figures 1B, C**). By contrast, butyrate concentrations were lower in the recovery samples than in the diarrhea samples, with no significant differences between the recovery and healthy samples (**Figure 1D**). We did not find a difference in histamine concentrations between diarrhea, recovery, and healthy samples (**Figure 1E**).

### Gut microbiota diversity

**Figure 2:**
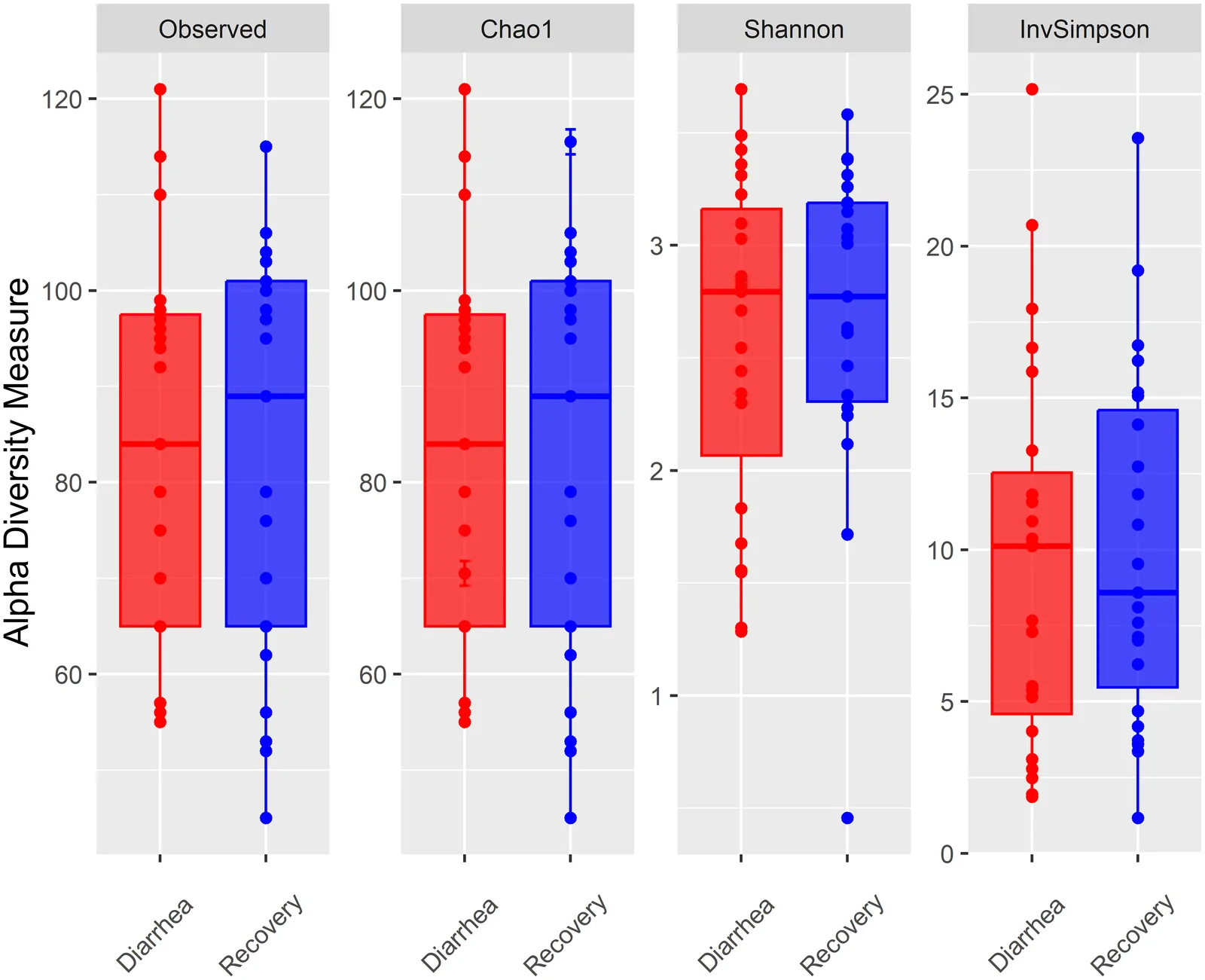
Alpha diversity index in the diarrhea and recovery samples. Observed, Chao1, Shannon, and InvSimpson alpha diversity values for the diarrhea (red) and recovery (blue) samples. A Wilcoxon signed-rank test for paired samples was performed to compare the two conditions.

**Figure 3:**
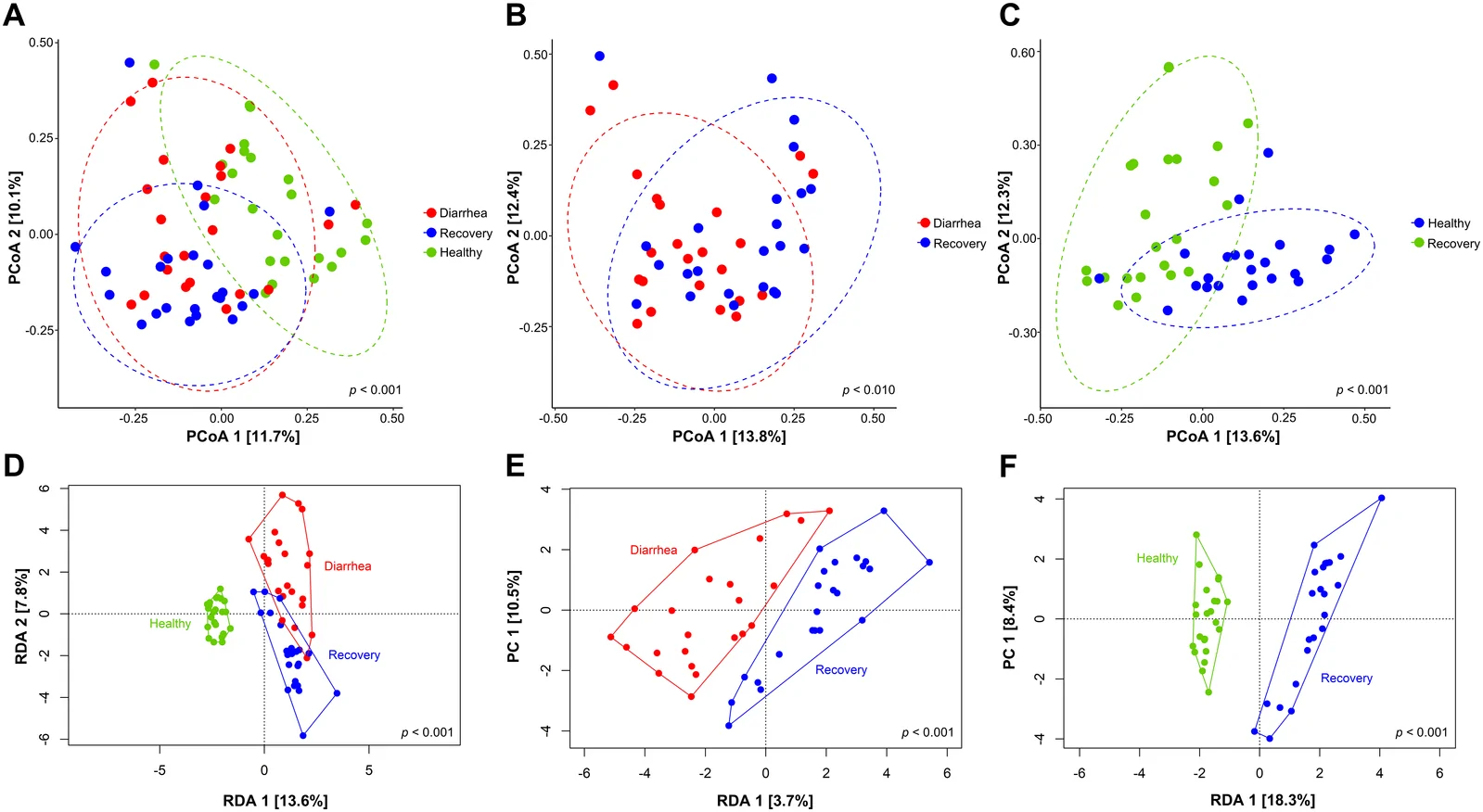
PCoA and RDA analysis of gut microbiota in the diarrhea, recovery, and healthy samples. PCoA plots **(A–C)** and RDA plots **(D–F)** based on the relative abundance of ASVs. Panels display comparisons among all three conditions **(A, D)**, between the diarrhea and recovery samples **(B, E)**, and between the recovery and healthy samples **(C, F)**. A paired-design PERMANOVA was employed to compare diarrhea and recovery samples, whereas a standard PERMANOVA was used to compare the healthy samples with the diarrhea and recovery samples.

No significant differences in alpha diversity were observed between diarrhea and recovery samples with the different indices analyzed (**Figure 2**). By contrast, the structure of the intestinal microbial community differed significantly among the study samples. PCoA analysis showed clustering within each condition, with significant differences between conditions by PERMANOVA (**Figure 3A**). Pairwise comparison, using a paired/design-aware analysis, revealed significant differences between the diarrhea and recovery samples (**Figure 3B**). The comparison between the recovery and healthy samples also showed significant differences (**Figure 3C**). RDA analysis confirms the PCoA results, showing distinct clustering within each condition (**Figure 3D**). Pairwise comparisons showed that the recovery samples differed significantly from the diarrhea samples (**Figure 3E**) and the healthy samples (**Figure 3F**). The differences noted persist even when children who received antibiotics or probiotics are excluded from the analysis (**Supplementary Figures 1A, B**).

### Gut microbiota composition

**Figure 4:**
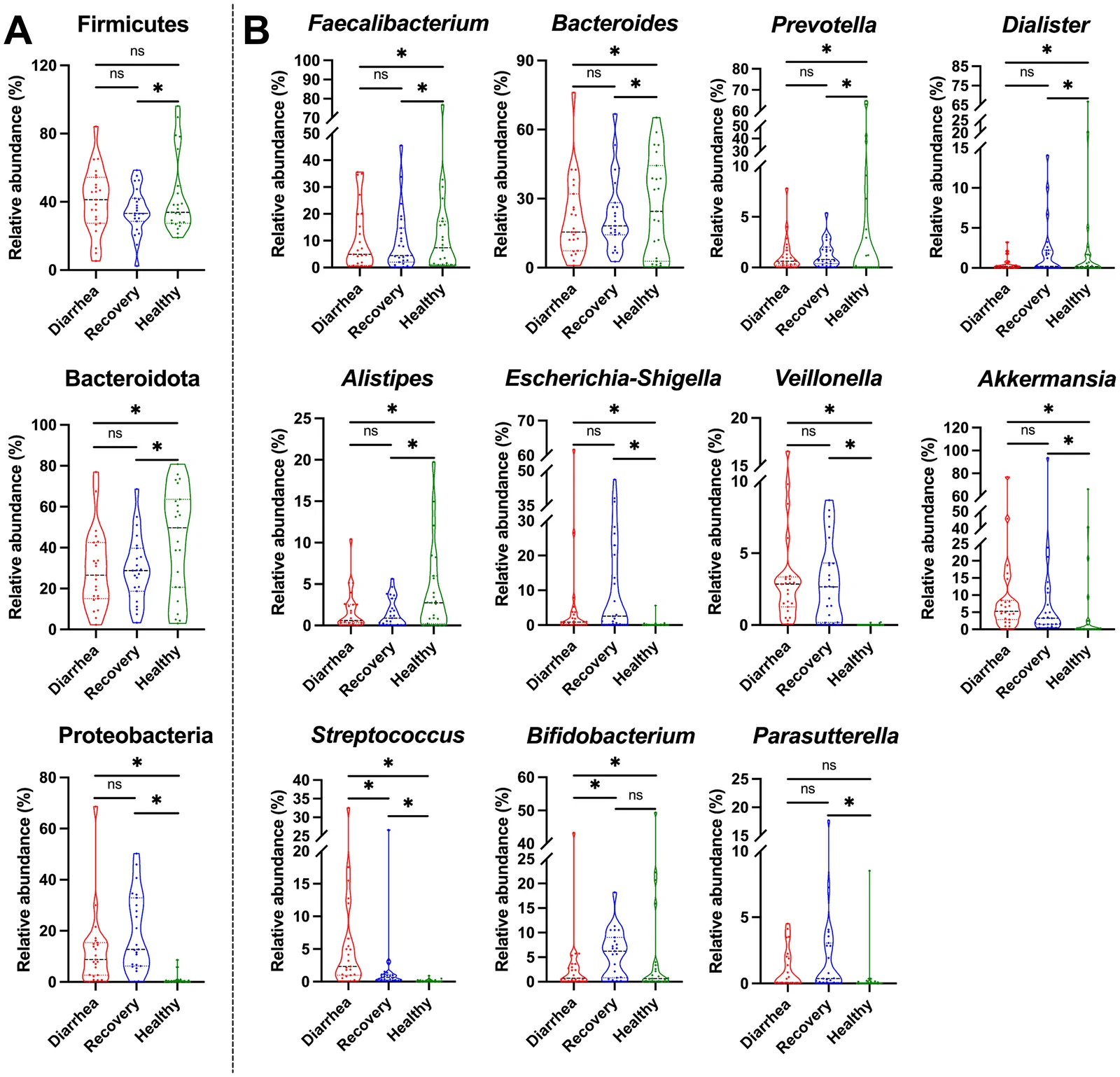
Relative abundance at different taxonomic levels in the diarrhea, recovery, and healthy samples. Relative bacterial abundance at the phylum **(A)** and genus **(B)** levels in fecal samples from children in the diarrhea, recovery, and healthy samples. A Wilcoxon matched-pairs signed-rank test was employed to compare the diarrhea and recovery samples, whereas an ANOVA followed by Tukey’s *post-hoc* test, with p-values adjusted for multiple comparisons using the False Discovery Rate (FDR) to compare the healthy samples with the diarrhea and recovery samples. Significance is indicated (*p<0.05).

We compared phylum-level abundances between samples. There were no differences between the diarrhea and recovery samples for Firmicutes, Bacteroidota, and Proteobacteria. Compared with the healthy samples, the diarrhea and recovery samples showed lower abundance of Bacteroidota, but higher abundance of Proteobacteria. For Firmicutes, a lower abundance was observed in the recovery samples compared to the healthy samples (**Figure 4A**).

At the genus level, we identified five patterns, each showing a significant difference between the study conditions (**Figure 4B**). The recovery and diarrhea samples showed significantly lower levels of *Faecalibacterium, Bacteroides, Prevotella, Dialister, and Alistipes* than the healthy samples (pattern 1). On the contrary, we found significantly higher levels of *Escherichia-Shigella*, *Veillonella, Akkermansia*, and *Streptococcus* in the diarrhea and recovery samples compared to healthy children (pattern 2); for *Streptococcus*, a significant difference was also found between diarrhea and recovery samples (pattern 3). In the case of *Bifidobacterium*, the diarrhea samples had lower levels of this genus than the healthy and recovery samples, but no significant differences were detected between the recovery and healthy samples (pattern 4). Finally, significantly higher levels of *Parasutterella* were observed in the recovery samples compared with the healthy samples (pattern 5).

**Figure 5:**
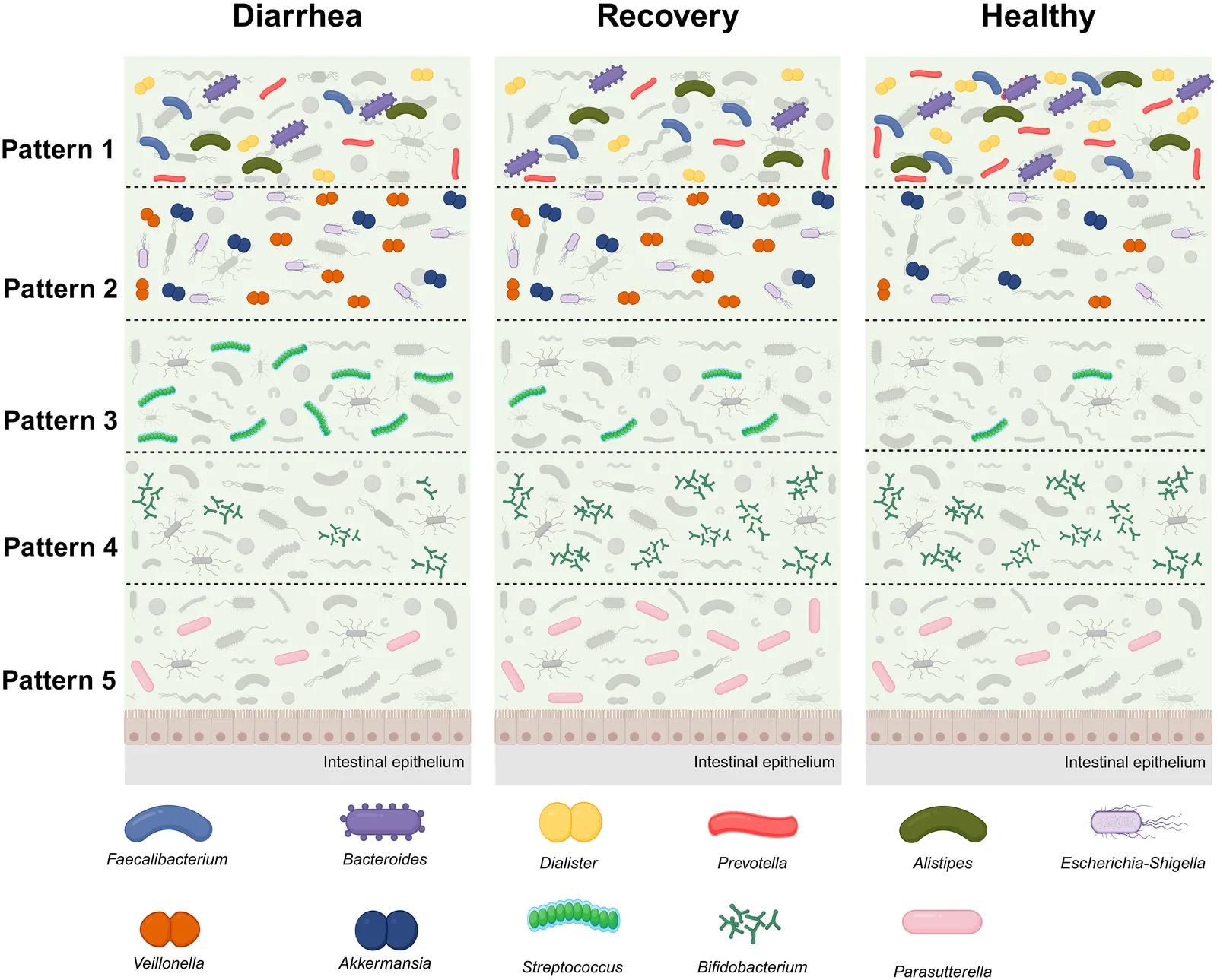
Proposed model of the pattern found in the study conditions, integrating the main findings of bacterial genera abundances. Pattern 1 reflects the levels of *Faecalibacterium, Bacteroides, Prevotella, Dialister*, and *Alistipes*. Pattern 2 reflects the levels of *Escherichia-Shigella*, *Veillonella*, and *Akkermansia.* Patterns 3, 4, and 5 reflect the levels of *Streptococcus, Bifidobacterium*, and *Parasutterella*, respectively. Created with BioRender.com.

For the remaining bacterial genera, no significant differences were found between recovery samples and either diarrhea or healthy samples. A model that integrates and summarizes all these patterns is presented in **Figure 5**.

## Discussion

In this study, we observed that the gut microbiota and metabolites associated with diarrhea in children who clinically recover from infectious diarrhea do not return to the levels observed in healthy children, while retaining similarities between the acute diarrheal and healthy conditions.

Butyrate decreased from the diarrheal phase to the recovery phase, reaching levels observed in healthy children, whereas total SCFAs, acetate, propionate, and histamine did not differ significantly among the conditions. However, across all metabolites tested, SCFA levels in the recovered children consistently fall between those of the diarrhea and healthy samples (**Figure 1**). These findings suggest a dissociation between metabolic and compositional recovery of the intestinal microbiota. Although other studies reported that diarrhea associated with *Campylobacter*, *Salmonella*, and *C. difficile* converges on similar deficiencies in intestinal microbial communities, including lower alpha diversity and reduced abundance of specific gut microbes (Singh et al., 2015), in our study, diarrhea caused by bacteria, viruses, or coinfections may have different impacts on the intestinal metabolome. Conversely, a metabolomic analysis in adults with diarrhea aged 19 to 64 years identified differences in metabolite diversity between fecal samples collected during diarrhea and after recovery (Hansen et al., 2024). Pathway prediction analyses showed that acetate synthesis pathways were preferentially associated with diarrhea, whereas butyrate synthesis pathways were associated with diarrhea recovery. In contrast, propionate-related pathways were not clearly linked to either condition. Our data revealed a post-recovery decrease in fecal butyrate, highlighting the distinction between predicted microbial genomic capacity and net metabolite excretion. Due to the lack of paired pediatric studies directly measuring post-diarrheal SCFAs, we compare our findings against adult metagenomic predictions (Hansen et al., 2024), which reported an enrichment of bacterial butyrate synthesis pathways during recovery. Crucially, fecal SCFA levels reflect the net balance between bacterial synthesis and host mucosal uptake. During acute diarrhea, mucosal inflammation impairs colonocyte butyrate transport, causing fecal accumulation. Upon clinical recovery, restored epithelial integrity and active consumption (For example, via MCT-1) normalize fecal butyrate concentrations despite ongoing microbial reorganization. Moreover, differences in population age, sampling time, and etiology are important factors that may contribute to the observed discrepancies. The stability of acetate and propionate levels across conditions aligns with the wide phylogenetic distribution of their producers (Louis et al., 2014), which, together with the absence of differences in histamine levels, suggests a balance between production and degradation pathways. This observation should be tested further.

The community structure of the recovery samples remained differentiated from both the diarrhea and healthy samples (**Figure 3**). Alpha diversity data between diarrhea and recovery samples did not show any significant differences (**Figure 2**). These observations contrast with other studies, in which alpha diversity decreases following the diarrheal event and subsequently increases over time, reaching levels of richness and diversity observed before the diarrheal episodes after 40–45 days (Mortensen et al., 2018) or 60 days (Becker-Dreps et al., 2015). In our study, despite a longer median follow-up period of 2.5 months, alpha diversity remains unrecovered, indicating that the ecological restoration of the gut microbiota may require an even longer time frame in this population. This sustained low diversity may be explained by two key factors; first, 52.2% of the clinically recovered children in our cohort still exhibit persistent pathogen detection (**Table 2**), suggesting that asymptomatic carriage maintains a state of low-grade ecological disruption. Second, in children <5 years of age the gut microbiota is still undergoing maturation, making its baseline structure highly dynamic and less resilient than that of adults. Thus, our data demonstrates that clinical resolution of gastrointestinal symptoms precedes the full recovery of microbial richness and diversity.

In the healthy gut, microbial communities are typically dominated by obligate anaerobes such as Firmicutes, while Proteobacteria are maintained at low relative abundance. During acute enteric infections and diarrheal episodes, intestinal inflammation disrupts this equilibrium, typically leading to a depletion of Firmicutes and an expansion of facultative anaerobic Proteobacteria (Kamada et al., 2013; Gallardo et al., 2017; Kho and Lal, 2018). Consistent with this pattern, in our cohort, Proteobacteria remained elevated in both diarrhea and recovery samples compared with healthy samples and were even higher in the recovery samples. However, we observed that Firmicutes abundance was lower in recovery samples than in healthy samples (**Figure 4A**). Similarly, genera associated with acute diarrheal episodes, such as *Escherichia-Shigella*, *Streptococcus, Veillonella*, and *Akkermansia*, remained relatively enriched in the diarrhea and recovery samples compared to the healthy samples (**Figure 4B**). This observation may be influenced by the detection of pathogens in recovery samples, where 52.2% tested positive. The persistence of Proteobacteria-dominated dysbiosis is particularly noteworthy given the pro-inflammatory nature of this phylum. Many members of Proteobacteria can sustain inflammation via LPS-TLR4 signaling (Shin et al., 2015; Vatanen et al., 2016), which may perpetuate dysbiosis beyond clinical recovery. Alongside similarities between the recovery and diarrhea samples, we also observed repopulation of genera associated with the healthy condition. For example, recovery samples showed an expansion of *Bifidobacterium* toward abundances observed in healthy children. The restoration of this genus is biologically plausible given its competitiveness and its cross-feeding role within SCFA networks (O'Callaghan and van Sinderen, 2016). Altogether, these data suggest that the population of genera in recovered children lies between those in healthy children and those in children with diarrhea.

Our study has several limitations. Although the study population is small, it exceeds the calculated sample size needed to assess whether the gut microbiota differs significantly among the three conditions. It is important to mention that this type of study is complex to conduct due to the study population and its high cost of enrollment and sample analysis. Regarding the study population, the inclusion criterion in this work was children with diarrhea who presented to the hospital and tested positive for one or more enteropathogens. Therefore, it is not possible to associate these findings with a particular enteropathogen. Regarding the recovery sample, the criterion was the absence of diarrhea-associated symptoms, even though enteropathogens were detected in some of them. The presence of enteropathogens in children without diarrhea symptoms has been widely described in other populations (Lima et al., 2019; Levine et al., 2020). The lack of pre-diarrheal samples and longitudinal follow-up after recovery from diarrhea prevents characterization of the trajectories of the intestinal microbiota and metabolome during clinical recovery and beyond the interval evaluated in this study.

In summary, clinical recovery from a diarrheal episode in children is accompanied by a normalization of certain fecal metabolites, particularly butyrate, while the community structure remains distinct among conditions. Moreover, similarities persist at the genus level during recovery, with a mixed profile between the acute diarrhea and healthy samples. Regarding the clinical impact, our results suggest that restoration of the microbiota and metabolome following an episode of diarrhea takes longer than in the absence of gastrointestinal symptoms, information that may be relevant for potential microbiota-based interventions for the therapeutic management of diarrhea.

## Data Availability

The datasets presented in this study can be found in online repositories. The names of the repository/repositories and accession number(s) can be found in the article/**Supplementary Material**. The 16S rDNA barcoding data were deposited in the NCBI Sequence Read Archive (SRA) database under accession PRJNA1335915.

## Supplementary material

Supplementary Figure 1 PCoA and RDA analysis of gut microbiota in children who did not receive antibiotic or probiotic treatment. Graphical representation of PCoA and RDA analysis of the gut microbiota in diarrhea, recovery, and healthy samples. Analysis restricted to the sub-group of children who did not receive antibiotic **(A)** or probiotic **(B)** treatment. Statistical significance between study samples within these subsets was assessed using PERMANOVA (p < 0.05).
