# Supplementary material for: Dynamics of the gut microbiome and metabolome in children during and after an infectious diarrheal episode
Source: Front Cell Infect Microbiol. 2026 Aug 27;16:1905670. doi: 10.3389/fcimb.2026.1905670 (PMC13557952; doi:10.3389/fcimb.2026.1905670)
Supplement: Supplementary file 1 [file Table1.docx]

| **Child** | **Sex** | **Age  (months)** | **Use of antibiotics** | **Use of antibiotics before diarrhea episode** | **Use of antibiotics for diarrhea treatment** | **Use of probiotics** | **Use of probiotics before diarrhea episode** | **Use of probiotics for diarrhea treatment** | **Days between diarrhea episodes and recovery sampling (months)** | **Type of delivery** | **Breastfeeding** | **Any types of Allergies** |
| --- | --- | --- | --- | --- | --- | --- | --- | --- | --- | --- | --- | --- |
| 1 | Male | 40 | No | No | No | No | No | No | 1.6 | Cesarean | Yes | No |
| 2 | Female | 7 | No | No | No | No | No | No | 1.9 | Vaginal | No | No |
| 3 | Female | 36 | No | No | No | Yes | Yes | No | 4.5 | Cesarean | Yes | No |
| 4 | Male | 8 | No | No | No | No | No | No | 1.7 | Vaginal | Yes | No |
| 5 | Male | 22 | Yes | No | Yes | No | No | No | 2.8 | Vaginal | Yes | No |
| 6 | Female | 2 | No | No | No | No | No | No | 2.0 | Vaginal | Yes | No |
| 7 | Male | 2 | No | No | No | Yes | No | Yes | 4.8 | Vaginal | Yes | Yes |
| 8 | Male | 15 | No | No | No | No | No | No | 1.8 | Cesarean | Yes | No |
| 9 | Female | 36 | Yes | Yes | No | Yes | No | Yes | 1.5 | Cesarean | Yes | Yes |
| 10 | Male | 9 | No | No | No | No | No | No | 2.8 | Vaginal | Yes | No |
| 11 | Male | 22 | No | No | No | No | No | No | 4.9 | Cesarean | Yes | No |
| 12 | Male | 12 | No | No | No | Yes | Yes | No | 3.0 | Vaginal | Yes | No |
| 13 | Male | 3 | No | No | No | No | No | No | 2.4 | Cesarean | Yes | No |
| 14 | Male | 13 | No | No | No | No | No | No | 1.5 | Cesarean | Yes | No |
| 15 | Female | 26 | No | No | No | No | No | No | 2.8 | Vaginal | Yes | No |
| 16 | Female | 55 | Yes | Yes | No | No | No | No | 5.3 | Cesarean | Not available | No |
| 17 | Female | 35 | No | No | No | No | No | No | 5.5 | Cesarean | Yes | No |
| 18 | Male | 13 | No | No | No | No | No | No | 3.1 | Cesarean | Yes | Yes |
| 19 | Male | 57 | Yes | No | Yes | No | No | No | 2.5 | Not available | Not available | No |
| 20 | Male | 51 | No | No | No | No | No | No | 1.0 | Vaginal | Yes | No |
| 21 | Female | 32 | Yes | No | Yes | No | No | No | 2.0 | Cesarean | Yes | No |
| 22 | Male | 50 | No | No | No | No | No | No | 1.1 | Cesarean | Yes | No |
| 23 | Male | 19 | No | No | No | No | No | No | 3.3 | Vaginal | Yes | No |

**Supplementary Table 1. Clinical data of enrolled patients in the diarrhea/recovery study.**
